# A hidden eddy-rich Antarctic shelf revealed by SWOT

**DOI:** 10.1093/nsr/nwag222

**Published:** 2026-04-14

**Authors:** Kay I Ohshima

**Affiliations:** Institute of Low Temperature Science, Hokkaido University, Japan

Antarctic continental shelves are key sites of water mass transformation, including dense shelf water (DSW) formation and ice-shelf melting, yet the role of mesoscale dynamics in mediating these processes has remained elusive due to severe observational limitations. Using the unprecedented spatial resolution of the Surface Water and Ocean Topography (SWOT) satellite, Han *et al.* [1] provide the first pan-Antarctic observational evidence of widespread mesoscale eddy activity, made possible by a step change in satellite altimetry that delivers two-dimensional, very high–resolution sea surface height measurements, thereby offering a new dynamical framework for understanding cross-shelf exchange in the Southern Ocean.

Antarctic continental shelves play a pivotal role in the global climate system. First, intense sea-ice production in coastal polynyas generates DSW through rejection of brine (salt-enriched water). This dense water contributes to the formation of Antarctic Bottom Water (AABW) [2], a key driver of the global overturning circulation. Second, ocean-driven basal melting of ice shelves regulates the mass balance of the Antarctic Ice Sheet and thus global sea level. Both processes inherently generate strong cross-shelf density gradients, which can give rise to baroclinic instability and mesoscale eddies [3]. Despite this theoretical expectation, direct observational evidence of such eddies across the Antarctic shelf has been lacking.

The SWOT-based analysis by Han *et al.* [1] reveals that Antarctic continental shelves are pervasively populated by mesoscale eddies. Enhanced eddy activity is particularly evident in regions of active DSW formation and ice-shelf melting. The characteristic eddy scale, on the order of ∼10 km, is significantly smaller than that in mid-latitude or open-ocean environments, consistent with the small Rossby deformation radius (∼5 km or less) associated with weak stratification and strong Coriolis effects in polar regions. Idealized numerical simulations further demonstrate that both DSW formation and ice-shelf melting can independently generate eddies with properties consistent with observations, providing strong mechanistic support for the proposed framework. From this perspective, the Antarctic shelf should no longer be viewed as a relatively quiescent boundary, but rather as a dynamically active system in which mesoscale eddies play a central role in mediating cross-shelf exchanges of heat and freshwater.

These findings suggest that SWOT observations could potentially be used to monitor ice-shelf melting and DSW export. Recent studies suggest a slowdown in AABW formation and export [2,4], although uncertainties remain. Further, ocean-driven basal melting of Antarctic ice shelves is accelerating [5], potentially enhancing surface freshening and thereby influencing AABW formation [6]. In this context, satellite-based observations of eddy activity may offer a novel pathway to track changes in key Antarctic processes and their climatic impacts.

Several important questions remain. While Han *et al.* [1] focus on the Ross Sea, other major DSW formation regions, such as off Adélie Land and Cape Darnley [7], warrant similar investigation. Because both DSW formation and ice-shelf melting originate in the ocean interior, combining SWOT observations with *in situ* measurements, such as moorings, will be essential to fully resolve the underlying dynamics. Sea-ice melt may also act as a distinct mechanism for eddy generation, and its contribution remains to be clarified. Moreover, the detection of sub-10 km eddies approaches the resolution limit of SWOT, highlighting the need for careful validation. A key limitation is that SWOT cannot currently retrieve sea surface height in ice-covered regions, particularly during winter when DSW formation is most active. Looking ahead, advances in sea-ice classification and lead detection—building on experience from conventional altimetry—may eventually enable SWOT to extend its capability into partially ice−covered regions, potentially allowing year-round monitoring of Antarctic shelf processes.
